# A tale of two densities: active inference is enactive inference

**Published:** 2019-07-21

**Authors:** Maxwell JD Ramstead, Michael D Kirchhoff, Karl J Friston

**Affiliations:** 1 Division of Social and Transcultural Psychiatry, Department of Psychiatry, McGill University, Montreal, QC, Canada; 2 Culture, Mind, and Brain Program, McGill University, Montreal, QC, Canada; 3 Department of Philosophy, McGill University, Montreal, QC, Canada; 4 Department of Philosophy, Faculty of Law, Humanities and the Arts, University of Wollongong, Wollongong, Australia; 5 Wellcome Centre for Human Neuroimaging, University College London, London, UK

**Keywords:** Active inference, free-energy principle, representationalism, enactivism, structural representations

## Abstract

The aim of this article is to clarify how best to interpret some of the central constructs that underwrite the free-energy principle (FEP) – and its corollary, active inference – in theoretical neuroscience and biology: namely, the role that generative models and variational densities play in this theory. We argue that these constructs have been systematically misrepresented in the literature, because of the conflation between the FEP and active inference, on the one hand, and distinct (albeit closely related) Bayesian formulations, centred on the brain – variously known as predictive processing, predictive coding or the prediction error minimisation framework. More specifically, we examine two contrasting interpretations of these models: a structural representationalist interpretation and an enactive interpretation. We argue that the structural representationalist interpretation of generative and recognition models does not do justice to the role that these constructs play in active inference under the FEP. We propose an enactive interpretation of active inference – what might be called *enactive inference*. In active inference under the FEP, the generative and recognition models are best cast as realising inference and control – the self-organising, belief-guided selection of action policies – and do not have the properties ascribed by structural representationalists.

## 1. Introduction

The aim of this article is to clarify how best to interpret some of the central constructs that underwrite the free-energy principle (FEP) – and its corollary, active inference – in theoretical neuroscience and biology: namely, the role that generative models and recognition densities^1^ play in this theory, aiming to unify life and mind (Friston, 2013; Kirchhoff, Parr, Palacios, Friston, & Kiverstein, 2018; Ramstead, Badcock, & Friston, 2018). We argue that these central constructs have been systematically misrepresented in the literature, because of the conflation between active inference, on the one hand, and distinct (albeit closely related) Bayesian formulations, centred on the brain – variously known as predictive processing (Clark, 2013, 2015; Metzinger & Wiese, 2017), predictive coding (Rao & Ballard, 1999) or the prediction error minimisation (PEM) framework (Kiefer & Hohwy, 2018, 2019).

These latter approaches have much in common with active inference, and together constitute what might be called Bayesian cognitive science. The idea behind these Bayesian approaches is, in a nutshell, that *cognitive processes are underwritten by predictions based on inferential models*. Central among these models are *generative models*– that is, statistical models of how sensory observations are generated, which harness the prior beliefs (i.e., probability densities) of a cognitive system about its environment. In Bayesian cognitive science, these generative models are said to work in tandem with *recognition models*– which harness posterior beliefs that represent the system’s observationally informed ‘best guess’ about the causes of its sensations. Bayesian schemes treat cognitive activity as inferring a posterior probability distribution (a guess about the causes of sensory states – the recognition density) via a process of belief updating – essentially, changing prior beliefs (from the generative model) into a posterior belief, by assimilating new observations or sensory evidence.

The question that shall occupy us is how best to understand the function and properties of the generative and recognition models *in active inference under the FEP*, in light of the active processes involved in orchestrating, maintaining and updating these models. In particular, we examine two contrasting interpretations of these models: a *structural representationalist* interpretation and an *enactive* interpretation.

Recent work on the Bayesian approach casts generative models (and associated recognition densities) as *structural representations*– that is, as ‘iconic representations in which the structure of internal representations in the brain come to replicate the structure of the generative process by which sensory input impinges upon it’ (Williams & Colling, 2017, p. 1962). The most engaging recent defence of structural representationalism, which will be our target, have been provided by Clark (2015), Gładziejewski (2016), Gładziejewski and Miłkowski (2017), Hohwy (2014, 2016), Kiefer and Hohwy (2018, 2019), Williams (2017) and Williams and Colling (2017). On this view, cognitive processes are seen as irreducibly involving internal, neural structures that carry representational content, and which acquire their contents via inferential processes in the hierarchical generative and recognition models that are instantiated by the brain.

We argue that the structural representationalist interpretation of generative and recognition models – while providing an accurate description of these constructs as they figure in some versions of Bayesian cognitive science – does not do justice to the generative models and recognition densities that figure *in active inference under the FEP*. In contrast to these other Bayesian theories, which are, in effect, theories of the structure, function and dynamics of the *brain*, active inference is a much broader theory of *adaptive phenotypes*, that centres on the *control of adaptive behaviour* and that emphasises the tight coupling and circular causality between perception and action.

The *enactive* interpretation of active inference that we pursue takes seriously the idea that active inference is a *self-organising process of action policy selection*. When understood as a self-organised policy selection, active inference has the following non-trivial implication. Active inference is not merely a view of the brain as reducing the uncertainty of its sensory observations via perceptual inference. It concerns the active, selective sampling of the world by an embodied agent. From a technical point of view, active inference and perceptual inference are not merely two sides of the same coin. Instead, active inference is the name of the formulation for policy selection. What advocates of the Bayesian brain call ‘perceptual inference’ is just one moment of the policy selection process in active inference under the FEP, namely, state estimation. The issue we want to press here is that the active inference framework implies that perception is a form of action, that is, action and perception cannot be pulled apart as they sometimes are in the Bayesian brain framework.

In this sense, the active inference scheme is enactive (Thompson, 2010; Varela, Thompson, & Rosch, 1991), in the enactive sense of being for action (Bruineberg & Rietveld, 2014; Kirchhoff, 2018; Kirchhoff & Froese, 2017; Kirchhoff & Kiverstein, 2019; Ramstead et al., 2018; Ramstead, Kirchhoff, Constant, & Friston, 2019). Our enactive interpretation of active inference – what might be called *enactive inference*– follows what has been called the pragmatic turn in cognitive science (Engel, Friston, & Kragic, 2016). In cognitive science, this is the move away from a view of cognition as the rule-governed manipulation of internal (often symbolic) representations, to a view of cognition as being essentially action-oriented, and therefore premised on the selection of adequate forms of situationally appropriate action.

We proceed differently from much of the literature discussing this question, in that we base our interpretation of generative and recognition models directly on the mathematical apparatus of active inference. Namely, we examine the FEP and active inference as applied to the selection of adaptive action policies – in contrast to other approaches that focus on the Bayesian *brain* and predictive coding, for example, Clark (2015) and Hohwy (2014). In active inference under the FEP, the generative and recognition models are best cast as realising *inference and control*– the belief-guided selection of action policies – and do not have the properties ascribed by structural representationalists. We thus provide a philosophical and information-theoretic justification for an enactive view of generative models under the FEP.

The argumentative structure of this article is as follows. In the first section, we present the generative and recognition models, as they figure in Bayesian cognitive science, and examine the claim that these inferential models are structural representations. In the second section, we present the FEP and active inference. In the third section, we examine in some detail the generative models and recognition densities that are featured in active inference under the FEP, emphasising the circular causality between action and perception that is implicit in these formulations. Finally, in the fourth section, we present the argument for enactive inference: generative models are control systems, and they are not structural representations.

## 2. Statistical models as representations

### 2.1. Generative models and recognition models in Bayesian cognitive science

Bayesian cognitive science is an approach to the study of cognitive systems that has gained much momentum in the last few decades (Ballard, Hinton, & Sejnowski, 1983; Friston, 2010; Rao & Ballard, 1999). On this approach, cognitive systems can be described as instantiating a form of Bayesian inference. That is, their physical properties and patterns of behaviour come to match (or infer, in a statistical sense) those of their embedding ecological niche (Bruineberg, Kiverstein, & Rietveld, 2016; Kiefer, 2017). The various flavours of Bayesian cognitive science – for example, the Bayesian brain (Knill & Pouget, 2004), predictive coding (Rao & Ballard, 1999) and active inference (Friston, 2010) – furnish mathematical tools to model how organisms engage with their worlds (Lee & Mumford, 2003; Mumford, 1992).

This framework is broadly *Bayesian* because it rests on the idea that, at some level of description, organisms encode expectations or beliefs about their environment, which guide their cognitive processes (Rao & Ballard, 1999). These beliefs have been formalised as *Bayesian posteriors and priors*. Bayesian priors in this context correspond to probability distributions that are parameterised or shaped by physical states, for example, brain states and patterns of neural activity.^2^ Bayes’ theorem tells us how to combine optimally what we know about the probability of some unobserved state or hypothesis *s*, prior to making any observation – that is, Bayesian *prior beliefs*, which is denoted P(s)– with what we know, *given* some data or sensory observation *o*– that is, *likelihoods*, denoted P(o|s). Bayes’ theorem tells us that the posterior probability of some event, *given* some sensory data, is proportional to the product of the prior and likelihood

$$P(s|o)=\frac{P(o|s)P(s)}{P(o)}$$

The Bayesian claim that will concern us can be stated more specifically as follows: cognitive systems act as if they are inferring the causes of their sensations, that is, inferring the most probable event or hypothesis, given the sensory observation.

This kind of anticipatory engagement evinces a role for *statistical models* (i.e., probability densities), based on which the relevant predictions can be made, and adaptive actions can be selected. If the organism has access to a *model* of what states are the most expected, statistically speaking, then it can compare its current state to this model, instead of trying to evaluate how surprised it is relative to all its possible states. Indeed, this evaluation, which involves computing the marginal likelihood or evidence P(o), often turns out to be an intractable problem (Friston, 2010; Kiefer & Hohwy, 2018). Most Bayesian schemes in cognitive science suggest that organismic dynamics can be described as *bounding* surprise by ‘guessing’ (i.e., approximating) how surprising their sensory states are, based on *statistical models* of their predicted sensations – hence the appeal to *approximate* Bayesian inference. These schemes are based implicitly or explicitly on optimising an evidence bound called variational free energy (Friston, Parr, & de Vries, 2017). We now briefly rehearse Bayesian inference to unpack these terms.

In Bayesian cognitive science, the *generative model* (that comprises a likelihood and prior density) is said to be *inverted* to give the *recognition model* (that constitutes a posterior density). A generative model is a probabilistic model, denoted P(o,s), of how sensory observations are generated. It is a statistical mapping from hidden causes *s*, which include external states of – or causes in – the environment to sensory observations *o*. Technically, the generative model is a joint probability distribution or density over hidden causes and observations. We work with generative models more easily when they are expressed in a form amenable to Bayesian parameterisation, as the product of *likelihood* and a *prior*

P(o,s)=P(o|s)P(s)

The beliefs harnessed in the recognition and generative models need to be updated to allow for adaptive cognitive processes. There are several ways to implement belief updating. In Bayesian approaches such as predictive coding (Rao & Ballard, 1999) and active inference (Friston, 2010), belief updating entails the formation of posterior beliefs about the causes of sensations, using approximate Bayesian inference. Technically, these (Bayesian) beliefs are referred to as *approximate posteriors, variational densities* or *recognition densities*. The *recognition model* is the inverse of a likelihood model: it is a statistical mapping from observable consequences to hidden causes. This explains why forming a posterior belief is often referred to as model inversion, where

Q(s)≈P(s|o)

In other words, the recognition model is an approximate posterior probability distribution or Bayesian belief that constitutes the organism’s ‘best guess’ about what is causing its sensory states (including the consequences of its own actions). It is called a recognition model because the model allows one to determine – that is, to recognise – the most likely cause of a given observation. In contemporary belief updating schemes, optimising beliefs involve minimising a quantity called *variational free energy*

$$\begin{array}{c} Q(s)=\arg\min_{Q}F(Q)\Rightarrow Q(s)\approx P(s|o)\\ F(Q)=E_{Q}[\ln Q(s)-\ln P(s,o)]\\ =E_{Q}[\ln Q(s)-\ln P(s|o)]-\ln P(o)\geq -\ln P(o) \end{array}$$

By construction, variational free energy F(Q)≥−lnP(s) is an upper bound on negative log evidence, which is also called *self-information* or *surprise* in information theory. This means that any system that avoids surprising exchanges with the world (i.e., surprising sensory states) will look as if it is predicting, tracking and minimising a quantity called *variational free energy*, on average and over time. Variational free energy quantifies the difference between what an organism *expects* to encounter and what it observes, where observations can be about exteroceptive, interoceptive or proprioceptive causes of input. In this sense, it can be thought of as some generalised prediction error. On this view, all the processes involved in cognition, from perception to learning and action, minimise the difference between expected sensory states (given prior beliefs) and observations, which gives them the look and feel of Bayesian inference.

This optimisation can proceed explicitly as in predictive coding (Rao & Ballard, 1999), belief propagation (Pearl, 1982) and (marginal) neuronal message passing (Parr, Markovic, Kiebel, & Friston, 2019) – depending upon the form of the general model and optimisation scheme. Some schemes try to learn a mapping from sensory inputs to the recognition density, assuming the parameters of this implicit recognition model do not change with time or context. This effectively converts an inference problem into a learning problem – as seen in earlier formulations like the Helmholtz machine (Dayan, Hinton, Neal, & Zemel, 1995). The more general theme – that underwrites approximate Bayesian inference – is that we can convert a mathematically intractable inference problem into an optimisation problem by extremizing variational free energy (e.g., by minimising prediction error). Once inference is cast as optimisation, one can then associate the dynamics of any sentient system (e.g., creatures like you and me) as implementing inference, via optimisation through a process known as gradient descent (Friston, 2013).

### 2.2. Generative models as structural representations

In this section, we unpack the notion that generative models are structural representations, which is the critical target of this article. We will focus on the most recent, compelling and engaging defence of this claim, provided by Kiefer and Hohwy (2018, 2019), Gładziejewski and Miłkowski (2017), and Gładziejewski (2016).

Generally speaking, *representations* are explanatory constructs that are posited in cognitive science to make sense of the capacity of a cognitive system to engage in intelligent action (Williams & Colling, 2017). In this literature, representations are defined as structures and associated dynamics that are internal to an organism – typically, states and processes of their nervous systems, especially their brains. What makes these structures special, and useful in explanation, is that they carry *representational content*, by virtue of which the organism is able to engage its ecological niche through adaptive behaviour (Boone & Piccinini, 2016; Ramsey, 2007). Representational content is what the representation is about –‘that is, in virtue of what they represent what they do, or get to be “about” what they are about’ (Kiefer & Hohwy, 2018, p. 2390).

An increasingly popular line of argument holds that the relevant neural structures function as *iconic* or *structural representations* that carry *structural content*. More specifically, structural representations operate via *exploitable structural similarity* (Gładziejewski, 2016; Gładziejewski & Miłkowski, 2017; Hohwy, 2014; Kiefer & Hohwy, 2018, 2019). On this account, structural representations get their representational contents (1) from their standing in a relation of *structural similarity* to the target domain, in the sense that the second-order structural features (e.g., statistical properties; O’Brien & Opie, 2004) of the target domain are recapitulated in, or mirrored by, those of the neural representation and (2) from being *exploitable* by the organism or agent, in the sense that the information about the target domain encoded in the neural states can be leveraged by the cognitive system to guide intelligent, adaptive behaviour. This exploitable similarity relation is weaker than strict isomorphism, and goes beyond mere resemblance in that it requires that the encoded second-level structural resemblance in question must be *causally relevant* to the behavioural success of the organism (Gładziejewski & Miłkowski, 2017; Williams & Colling, 2017). Structural representations are also described (3) as *detachable*, in the sense that they can be used by the agent to perform cognitive tasks ‘offline’, and (4) as *affording representational error detection*– in a manner analogous to cartographic maps – which allows for coupled adaptive action in the world. This last clause specifies what is at stake in (1) and (2): representational error, here, refers to the idea that the *user* of representation can ‘get it wrong’. The structural representation, like the map, does not itself afford representational error – its use by the system does.

Recent defences of structural representations in theoretical neuroscience have leveraged the resources of the PEM framework to argue that the *generative models* that figure in Bayesian approaches to cognitive science are *structural representations*. That is, proponents of structural representations argue that generative models function as structural representations with representational content. A great summary of this view reads that predictive coding theory

postulates internal structures whose functioning inside a cognitive system closely resembles the functioning of cartographic maps. It might be said that on the proposed interpretation of the theory, cognitive systems navigate their actions through the use of a sort of causal–probabilistic “maps” of the world. These maps play the role of representations within the theory. Specifically, *this map-like role is played by the generative models*. It is *generative models* that, similarly to maps, *constitute action-guiding, detachable, structural representations that afford representational error detection*. (Gładziejewski, 2016, p. 569, emphasis added)

The claim, then, is that generative models are structural representations, which are implemented by the exploitable structure and dynamics of neural networks in the brain: ‘This generative model can be understood as a sort of brain-implemented statistical or Bayesian network . . . whose structure resembles the causal-probabilistic structure of our system’s environment’ (Gładziejewski, 2016, p. 571). So, in summary, on this reading, generative models are neural structures that represent, stand in for, or act as proxies for states of affairs outside the brain in virtue of an exploitable structural similarity.

Kiefer and Hohwy (2018, 2019) examine the way that the generative model and recognition model constructs have been used in some Bayesian cognitive science. They focus on versions of Bayesian cognitive science that leverage the variational formalism, namely Bishop’s (2006) variational approach to machine learning. Summarising their view elegantly, they write:

as priors and likelihoods of hypotheses are mutually adjusted in light of prediction error, a reliable channel of information transmission is set up between neural populations encoding sensory input and higher-level representations – an approximate recognition model. In the other direction, a reliable channel is also constructed from those high level representations back down to the sensory input layers – the generative model. Since sensory input drives a signal up through the hierarchy, which reaches the highest levels, and then those high-level representations send signals back down through the hierarchy to the lowest levels, we can think of the overall network as learning a mapping from sensory input, through high-level representations of causes, back onto sensory input. (Kiefer & Hohwy, 2018, p. 2405)

We believe this to be an articulate description of *non-enactive* appeals to the Bayesian brain and variational Bayesian principles. The outstanding question for us is whether this view of the generative model accurately describes these constructs as they are used *in active inference under the FEP*.

## 3. The active inference framework

### 3.1. Phenotypes and Markov blankets

Living systems are unique in nature, since among all self-organising systems, they seem to maintain their organisation when facing environmental perturbations. Most self-organising systems dissipate the gradients around which they emerge: a lightning bolt, for instance, effectively destroys the gradient in electrical charge that gave rise to it. Organisms, strikingly, not only self-organise but manage to persist across time as self-organising systems (Ramstead et al., 2018). Heuristically, we can say that organisms expect to be in their characteristic phenotypic states; surprising deviations from these expectations must be avoided to maintain the system within viable (i.e., phenotypic) states. The FEP leverages variational inference to describe the dynamics within this space of states that can be cast in terms of active inference and self-evidencing (Friston, Mattout, & Kilner, 2011; Friston, 2010; Hohwy, 2016).

Variational methods allow us to do more than model brain dynamics. Recently, it has been argued that they allow us to cast living systems and their phenotypes as statistical constructs, in the following sense (Friston, 2010; Ramstead et al., 2018). The system tends towards occupying those states on average and over time – they are literally ‘attracted’ to these states, in virtue of their flow that is necessary to counter the dispersive effects of random fluctuations (i.e., to resist entropic erosion). (Technically, the characteristic states of an organism constitute a random dynamical attractor.) This means that phenotypic states are frequented with a higher probability than others. It follows directly from this observation that the probability density over the space of possible states of an organism must have low entropy or spread.

**Figure 1.**
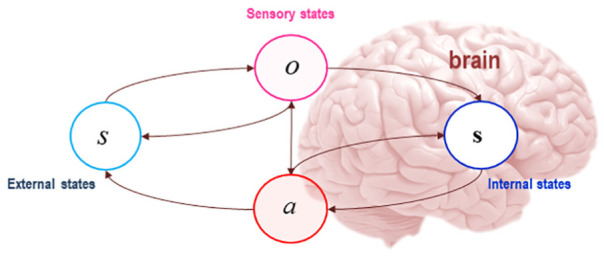
The Markov blanket and active inference. A Markov blanket is a set of states that isolates the internal states of a system, **s**, from external or hidden states *s*, in a statistical sense (for notational consistency, external states are italicised, while internal states are in boldface). In graph theoretic terms, the Markov blanket per se is defined as that set of nodes that isolates internal nodes from the influence of external ones, which means that external states can only affect internal states indirectly, via their effects on blanket states (Friston, Parr, & de Vries, 2017). The Markov blanket per se is made up of sensory states, which are denoted by *o*, and active states, denoted by *a*. Source: Adapted from Ramstead, Constant, Badcock, and Friston (2019). Figure re-used from REF under the CC license.

Active inference adds to the technical apparatus of variational inference the consideration of *Markov blankets* (Friston, 2013; Kirchhoff et al., 2018; Ramstead et al., 2018). The Markov blanket formalism provides an answer to the question: what counts as a system? A Markov blanket is a set of states that ‘enshrouds’ or isolates the system of interest in a statistical sense (see Figure 1). The presence of a Markov blanket partitions the whole system being studied (in our case, living systems engaging with their environmental niche) into internal (or systemic) states and external (environmental) states. The blanket itself can be partitioned into active and sensory states, which are defined as follows: active states are not influenced by external states, and sensory states are not influenced by internal states. The characteristic set or phenotype is then the set of expected or most probable states that constitute the system of interest; namely, internal states and their blanket.

### 3.2. Surprise, entropy and variational free energy

The FEP rests on a connection between three quantities in the context of Markov blankets: *surprisal, entropy* and *variational free energy*. The quantity called surprisal (or more simply, surprise) is quantity from information theory, which is a function of sensory states of the organism and measures the unexpectedness of a given state, namely, the (negative log) probability of a given sensory state being sampled. Under mild (ergodic) assumptions, the time average of surprise is equivalent to entropy (Friston, 2010). That is, assuming the system in question has robust features that can be measured more than once (i.e., that it possesses a random dynamical attractor), the average of surprise over time is their entropy H=E[−lnP(o)] (Ao, 2005, 2008; Seifert, 2012). Entropy in this context is a measure of the spread, dispersion or dissipation of systemic states; low entropy means that the system will occupy a limited number of states, compared to all possible states it could be in.

Crucially, the Markov blanket dynamics can be formulated entirely in terms of a gradient descent on surprise. Heuristically, this means that, so long as the Markov blanket is in play, the system must move necessarily towards the set of least surprising states – to exactly balance the dispersive effects of random fluctuations. Note that this means that the necessary conditions on the existence of a system (i.e., a Markov blanket) can be captured purely in terms of *surprise*.

*Variational free energy* gets into the game rather late in active inference: as noted above, organisms cannot measure the entropy of their states, nor how ‘surprising’ they are in any absolute sense – they are ‘just in’ a surprising state or not. To ‘know if’ states were surprising, they would need to evaluate an intractable number of possible states of being. In other words, they would need to evaluate all the possible states that they can be in (which is a truly massive number of states, given how many parts and configurations even a simple organism can comprise), and how surprising their current state is relative to all those possible states. This feat is, for the most part, computationally intractable (for technical details, see Friston, 2010) – it either cannot be accomplished or cannot in a biologically realistic timeframe by biologically plausible mechanisms. However, we can interpret the gradient flows implied by the existence of a Markov blanket in terms of a gradient descent on variational free energy, thereby equipping the dynamics with an inferential interpretation (and associated information geometry) in terms of approximate Bayesian inference. The key move behind this interpretation rests on associating the internal states with beliefs *about* external states, via the recognition density

$$\begin{array}{c} Q(s)\equiv Q_{s}(s)\\ s=\arg\min_{s}F(Q_{s})\Rightarrow Q_{s}(s)\approx P(s|o) \end{array}$$

In other words, we treat the internal states as parameterising beliefs *about* external states. This converts approximate Bayesian inference into an optimisation problem that is ‘solved’ by the dynamics of internal states, given sensory states of the Markov blanket.

Heuristically, variational free energy is a measure of surprise, that is often cast in terms of prediction error – namely, the difference between what *would be* the case, conditional on the organism’s ‘best guess’ about what caused its sensory states, and what it *does* observe. The concrete, material states and processes of an organism, in a sense, *embody* this guess. Unlike surprise, which only depends on states which the organism cannot access directly (the state of its Markov blanket *and* the state of the external world), the free energy is a function of the beliefs and *expectations* of an organism, that is, a function of Bayesian beliefs encoded by internal states.

### 3.3. Active inference: variational free energy and inferential models

In short, given a Markov blanket partition, it is fairly straightforward to show that internal states can be interpreted as encoding *Bayesian beliefs* about external states that cause its sensory states – and so play a central role in the construction of free energy, which is defined relative to these beliefs (Friston, 2013; Friston, 2010). The causes of sensory states are *hidden* from the internal states, ‘under’ or ‘behind’ the Markov blanket, given that sensory and active states separate internal and external states from one another (in a statistical sense).

To minimise or bound free energy means that the organism is optimising its expectations about (i.e., its Bayesian beliefs over) things veiled ‘behind’ a Markov blanket. When these expectations coincide with the actual posterior probability over external states, the variational free energy becomes equivalent to surprise. When they do not, free energy acts as a proxy (an upper bound) on surprise, in the sense that free energy will always be greater than surprise (Friston, 2012). This also makes free energy a bound on (negative) model evidence, because surprise is negative model evidence in Bayesian statistics.

The partitioning rule – based on the dependencies induced by a Markov blanket – induces a simple form of *active inference* (Friston et al., 2011; Friston, Kilner, & Harrison, 2006), in virtue of minimising surprise directly via a gradient flow (i.e., the flow towards the least surprising states). This is a way of saying that internal and active states are directly involved in maintaining the integrity of systemic boundaries: namely, the Markov blanket. Active inference, in its basic rendition, describes the tendency of dynamical systems – such as cognitive systems – to implement a dynamics that minimises (on average) their surprise, via perception and embodied activity in the world. Active inference captures the idea that this stipulative minimisation is instantiated in a *generative model* and realised through *adaptive action* (understood as the enactment of policies that minimise expected free energy).

In active inference, tracking and minimisation of *expected* free energy^3^ is a strategy that living systems may use in order to select adaptive actions. Regardless of the metaphysical status of free energy, *if an organism embodies the belief that its actions minimise free energy*, and *if it can select actions on its basis*, then that quantity has *physically real effects*– by virtue of its effects the action-guiding beliefs of organisms (i.e., policies). The expected free energy gives the organism the capacity to test the viability of its beliefs, since it tracks discrepancies between those beliefs and the way things turn out. In short, it is the beliefs about expected free energy that drives the selection of action policies. Organisms that are equipped with generative models of the causes and consequences of their action can exploit the free energy construct and use it to their advantage. Organisms self-organise to reap the benefits of variational free energy, giving their behaviour *appearing to resist the second law of thermodynamics*, according to which entropy must always globally increase (or, more precisely, of appearing to resist the *fluctuation theorem* that generalises the second law to open systems in nonequilibrium steady state).

An important distinction between active inference and the Bayesian brain is implicit in the selection of actions. This follows because this process of selection rests upon posterior beliefs about policies: namely, how to sample the environment to solicit observations. In other words, something new has been brought to the table – posterior beliefs about the external states and *actions upon those states*. Defenders of structural representational interpretations of the FEP, of course, do also acknowledge the role of action in the scheme (see, for example, Williams & Colling, 2017). However, as we shall discuss below, their representational gloss on the issue is not mandated by the mathematical framework that underwrites the FEP.

## 4. A tale of two densities: the generative model and recognition density under the FEP

### 4.1. The generative model and generative process in active inference

In this section, we provide the interpretation of generative models and recognition densities that is in play in active inference. The idea behind active inference, its Bayesian nature – and the reason it is considered a form of inference – is that the dynamics of living systems can be described as implicitly realising approximate Bayesian (i.e., variational) inference through the selection of adaptive action policies. Under the FEP, living systems can be regarded as instantiating a statistical (generative) model of their sensory exchanges with the ecological niche by realising a dynamics that bounds variational free energy.

Variational free energy, and its minimisation in active inference, depends on two quantities to which the living system has access: its sensory states (or observations), and the internal states ‘covered’ by its Markov blanket. The organism can optimise these quantities by leveraging two probability densities that it entails and embodies, respectively. These are the *generative model* and the *recognition model*. These two probability densities have a specific form and function under the FEP. Under the FEP, generative models are *not explicitly encoded* by physical states. That is, they are *not encoded by states of the brain*. Rather, it is the adaptive behaviour of the system that implements or instantiates a generative model. This is a crucial point that differentiates active inference from non-enactive appeals to the Bayesian brain. The generative model is *enacted*; in the sense that adaptive behaviour brings forth the conditional dependences captured by the generative model, that is, keeping the organism within its phenotypic, characteristic states.

**Figure 2.**
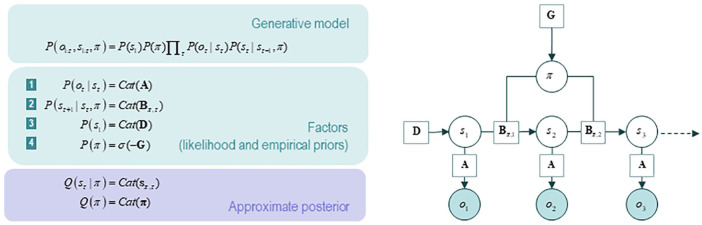
A generative model in active inference, represented as a Bayesian network. *Left panel*: Specification of the generative model. Technically, a generative model *P*(*o, s,π*) expresses the joint probability of sensory observations *o* and their causes *s, π*– where *s* denotes hidden states and *π* denotes the policy selected. A policy is just a sequence of active states, from which the next action is sampled. The model typically comprises a likelihood term (the probability of making a given observation, given causes) and prior beliefs about the hidden causes. In this model, the likelihood is specified by a matrix **A**, which captures the probability associated with a given outcome under every possible combination of causes. *Cat* denotes a categorical probability distribution. Empirical priors (priors that depend on variables) relate to transitions between hidden states, which are encoded in the **B** matrix. Hidden states, in turn, crucially include the actions of an organism, which are determined by policies. Prior preferences over outcomes are encoded in the **C** matrix, and the uncertainty or ambiguity associated with outcomes given each state are encoded by the **H** matrix. The vector **D** specifies the initial state. This generative model is constructed for policy selection; policies will be selected if they are more probable *a priori*; that is, if they minimise expected free energy *G*. The model is used to perform Bayesian model inversion. This is essentially the process of constructing a recognition density – an approximate posterior probability density that inverts generative mapping from consequences to causes, allowing for recognition based on observations (i.e., inferring the causes of sensory outcomes). *Right panel*: The generative model expressed as a Bayesian network. Such a network is a representation of the conditional dependencies between the causes of sensory outcomes. Open circles denote random variables, which must be inferred (i.e., hidden states and policies); filled circles denote observations. Squares denote known variables, such as the model parameters. Source: From Friston, Parr, and de Vries (2017). Figure re-used from REF under the CC license.

**Figure 3.**
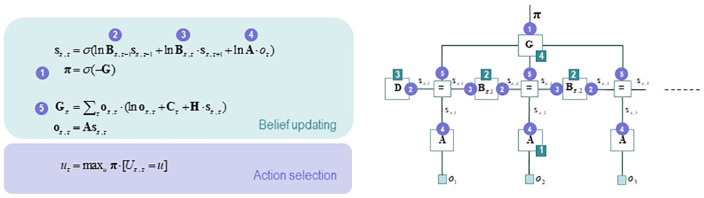
The same generative model in active inference, represented as a Forney factor graph. *Left panel*: Expressions for the belief updates enabling approximate Bayesian inference and action selection. In this figure, boldface denotes the expectations or sufficient statistics of hidden states in the previous figures. The brackets that figure in the action selection panel are Iverson brackets; if the condition in square brackets is obtained, these return the value 1, and return 0 otherwise. *Right panel*: Forney or normal style factor graphs are equivalent to Bayesian networks, with some important difference. In this kind of graph, nodes (the square boxes) correspond not to variables, as in a Bayesian network, but to factors; and edges represent unknown variables that must be inferred. Filled squares, echoing the above, denote observable outcomes. Edges are labelled in terms of the sufficient statistics of their marginal posteriors. Factors are labelled according to the parameters that encode the associated probability distributions. Circled numbers denote the implicit message passing in the belief updates – as messages are passed from nodes (factors) to edges (variables). Figure re-used from REF under the CC license. Source: From Friston, Parr, and de Vries (2017).

The technical term used in the literature for this realisation of a generative model is ‘entailment’ (Friston, 2012) – and refers to the fact that the statistical model in question is a consequence of the adaptive behaviour of the organism. Technically, the *dynamics* (i.e., the action policy selection) of a system are said to *entail a generative model* when the system is organised to actively instantiate (through active inference) a pair of probability density functions.^4^ These are the *recognition model* and the *generative model* per se. What this means, heuristically, is that the internal states entertain specific statistical relations to one another, such that they can be described as *realising the inversion* of a generative model. A generic generative model for policy selection is depicted in Figure 2 (as a Bayesian network) and in Figure 3 (as a Forney-style factor graph).

### 4.2. Variational inference and recognition dynamics under the FEP

Variational inference gets into the game because approximating the statistical structure of the environment involves guesswork and a few mathematical tricks, as it were. The organism does not have direct, unmitigated access to the generative process that produces its sensory observations. The organism only has access to the sensory states of its Markov blanket (i.e., to its sensory observations). In short, creating attractors in the joint space of ourselves and the environment is essentially a game of inference that is necessarily a game of probability and information as well.

Mathematically, in active inference, the *recognition density* operates as an arbitrary probability density function – over external (hidden) states – that is parameterised by the values of internal states. The recognition density itself is defined under the generative model. That is, the value of internal states encodes information that changes the form of the recognition density (changes the ‘guess’). In active inference, through the realisation of a free energy bounding dynamics, the recognition density embodied by the organism comes to approximate the sufficient statistics of the generative process from whence the creature emerged. The dynamics enacted in active inference is therefore equivalent to variational inference, what one might call a process of ‘embodied inference’ (Allen & Friston, 2016; Friston, 2011; Gallagher & Allen, 2016). Since the internal states of the Markov blanket are those states that constitute the system, we can think of the extended phenotype of the organism as literally *embodying or encoding* information that parameterises a recognition density.

Posterior densities over external states are approximated by tuning the internal states of the Markov blanket. The internal states encode the parameters of the recognition density (in terms of its sufficient statistics), which is the organism’s ‘best guess’ about what causes its observations. These parameters are optimised with respect to a variational bound on Bayesian model evidence. This bound *is* the variational free energy. This means that by tuning expectations about the cases of sensory data to bound free energy, the organism is also maximising evidence for a *statistical model* of its own existence (Friston, 2010).

‘Entailment’, in this setting, is used to emphasise that a generative model is necessary to define the recognition model but does *not* have sufficient statistics that are physically realised. In other words, a generative model is defined stipulatively as a probabilistic belief that explains the realised recognition model (i.e., perception and cognition) and subsequent action (i.e., policy selection and behaviour). See Friston (2012) for a formal treatment of entailment. Thus, the generative model is *entailed* by the internal dynamics, while the internal states encode the recognition model, in terms of sufficient statistics (e.g., expectations and precisions). The ‘causal bite’ of the generative model comes from the fact that it plays a role in policy selection by inducing free energy gradients (which then guide changes to beliefs about action). In other words, generative models are normative models of ‘what ought to be the case, given the kind of creature that I am’– they are realised physically through adaptive, belief-guided, normative actions that maintain the creature in its phenotypic states.

In summary, in active inference under the FEP, the generative model underwrites the selection of *adaptive action policies*. We can think of active inference as a story about how these two densities, the *generative and the recognition densities*, interact and change, and are *leveraged by the organism to engage in adaptive behaviour – a tale of two densities*, as it were. Our enactive interpretation proposes that changes in the recognition density, that is, alterations in the physical structure of the embodied organism, are controlled by the generative model, which selects which action policies to pursue on the basis of expected free energy. In this process of attunement, organisms change their structure (through learning and perception) and the structure of the world (through action), such that they become consistent with the preferences and expectations about the world that constitute the generative model (Bruineberg et al., 2016). In so doing, the generative and recognition models become attuned to the statistical structure of the environment from whence they emerged (i.e., the generative process).

## 5. Enactive inference

In this section, we unpack the implications of the pragmatist view for understanding the relations between the generative model, the generative process and the recognition model. According to our pragmatist interpretation, the organism *embodies* the recognition density and *entails* the generative model as a *control system*. We then formulate a direct critique of the claim that generative models are structural representations. We claim that to examine the role of generative models under the FEP makes it clear that they are necessarily distinct from the structures that encode or embody information about structural resemblance (i.e., the internal states). Simply put, on the assumption that proponents of structural representations are correct to claim that there are indeed physical structures that have the properties of structural representations under the FEP, they are incorrect to claim that the structures they identify as representations are generative models.

### 5.1. Generative models are control systems

A *generative process* couples the generative model of an organism to its environment, in a causally circular embrace reminiscent of the perception-action cycle (Fuster, 2004; Tishby & Polani, 2011). The generative process is what enables the generation of observations, enforcing the view that perception is non-trivially dependent on action. It is these observations to which an agent has ‘access’ to at any given time. The generative model is a statistical model of the generative process. Crucially, however, the generative model is distinct from the generative process (Friston, FitzGerald, Rigoli, Schwartenbeck, & Pezzulo, 2016). This is because the actual causes of sensory input depend on action (i.e., on a generative process), while action depends on inference (i.e., on a generative model).

This means that active inference depends on priors that inform action, while action per se affects the hidden causes generating sensory states (observations). In this formal sense, the function of the generative model is to couple the organism to its embedding environment via the generative process, which, in turn, completes the perception-action cycle.

Following Friston (2010), Seth (2014) and Anderson (2017), we now argue that, under active inference, the *generative model* functions as a *control system*. The organism uses its generative model to operate policy selection, the effect of which is to keep the organism within its phenotypic bounds (i.e., the organism’s phylogenetically and ontogenetically specified set points). Living systems exist in virtue of attaining nonequilibrium steady state (for some period of time); their dynamics do not resolve themselves through a return to thermal equilibrium states (i.e., death), but rather by the restoration of the system to a set of *attracting states* or set points (e.g., updates of the recognition model embodied by the organism).

This is key to understanding active inference. Active inference *generalises* approximate Bayesian inference, since in active inference the objective is not simply to infer the hidden states that cause observations but, more importantly, to act in such a way that minimises self-information or surprise (via minimising free energy) or minimises expected surprise or uncertainty (by minimising expected free energy). The reason for this is simple: active inference turns on the idea that it is *action*, upon which perception depends, that ultimately minimises uncertainty about the external causes of sensory observations. Hence, action can be cast as placing an upper bound on surprise – and expected surprise or uncertainty.

This is an important distinction between active inference and non-pragmatist appeals to a Bayesian brain hypothesis (e.g., predictive coding). In active inference, the inference is about sensory samples that are generated via action. In other words, the self-evidencing system is the author of its own sensations. This has the remarkable consequence (which we will appeal to later) that the generative model (in particular, prior beliefs) does all the heavy lifting in terms of structuring exchange with the environment. In other words, in most instances, the generative model is more deeply structured than the generative process describing the environment (unless we are engaging with someone else). This is particularly true for simple things like movement. There is nothing ‘out there’ that corresponds to the articulated movement of our hands, until it is authored by the organism.^5^

On this view, active inference can be read as a new take on *the good regulator theorem* proposed by Conant and Ross Ashby (1970) (see Friston, 2010). Active inference tells us about the relation between a *control system* (the generative model, with priors over action policies) and a system being controlled (the organism and its *adaptive behaviour*, the actual actions undertaken in, and part of, the world). This follows from a pragmatist reinterpretation of the good regulator theorem of Conant and Ashby. According to the good regulator theorem, one system can effectively *control* another if and only if that system is isomorphic with respect to the fundamental property of the system that it regulates, that is, if and only if it is a statistical model of the relevant properties of that system (Conant and Ross Ashby, 1970). The generative models mirror the structure of the generative process in order to control the behaviour of the organism. As such, generative models are more about the control and regulation of action than they are about figuring out what is ‘out there’ beyond the veil of sensory impressions, and representing the world (Anderson, 2017; Bruineberg et al., 2016). They enable survival, rather than tracking truth. They model the acting organism, and are *used* by living systems to modulate their behaviour.

The role of the generative model is to guide action in a contextually sensitive manner, which signals that we ought ‘to shift our focus from how brain mechanisms like Bayesian predictive coding implement and maintain models of the world, to how such mechanisms enable the feedback loops that maintain attunement to the environment and support adaptive behaviour’ (Anderson, 2017, p. 8). The generative model is vicariously realised – that is, brought forth or enacted – by the organism in active inference; the dynamics that is guided by the generative model integrates the partial contributions of model parameters embodied across spatial and temporal scales. The attunement of the generative model to the generative process is an indirect process that depends on the direct tuning of the recognition density embodied by the organism.

In summary, the role of a generative model is subtle in active inference. The generative model itself never actually exists outside the dynamics – that is, outside the adaptive actions and policy selection of the organism. Within the dynamics, it provides a point of reference or definition of variational free energy (or more precisely, a definition of the gradients with respect to internal and active states). Given that the vicarious realisation of the generative model (through a minimisation of variational free energy) can only be through action (and changes in internal states), we can think of the generative model as being *enacted*, and of the recognition density as being *embodied*.

This speaks directly to embodied and enactive approaches in cognitive neuroscience, and provides a computationally tractable framework for the metaphors mobilised by these paradigms (e.g., Gallagher, 2017; Noë, 2004; Thompson, 2010; Varela et al., 1991). The notion of entailment captures the fact that the generative model is entailed by the dynamics of a living system under active inference (Kirchhoff, 2018; Kirchhoff & Froese, 2017; Kirchhoff & Robertson, 2018; Ramstead, Kirchhoff, et al., 2019). This interpretation of the generative and recognition models allows us to model the dialectic between *embodiment* (what an organism *is*) and *enactment* (what an organism *does*). The generative model *is what the organism expects*, and *guides what the organism is and does*. It is constituted by expectations about the consequences of action that are conditioned upon the adaptive preferences of the organism. The recognition model *is the embodied organism*, in the sense that the physical states of the organism parameterise (embody or encode the parameters or *sufficient statistics* of) this density. Thus, the organism literally *embodies* the recognition model, and its patterns of action and perception *enact the expectations* of the generative model it *entails*. This interpretation allows us to evaluate and nuance representationalist conceptions of generative models under the FEP.

### 5.2. Generative models are not structural representations

**Figure 4.**
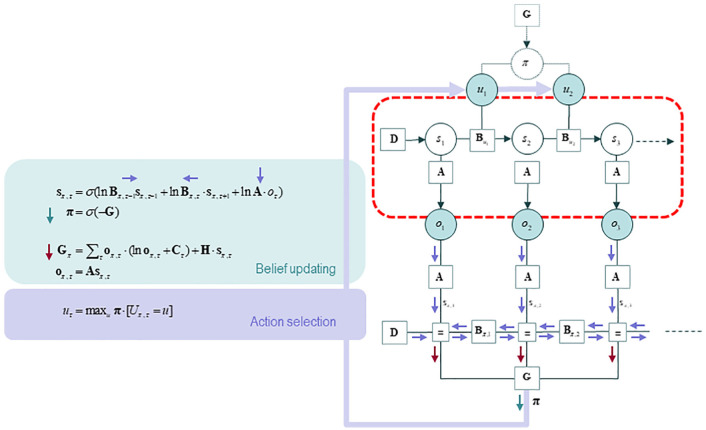
The action-perception cycle in active inference: A generative model and process. This figure combines the Bayesian network in Figure 2 and the Forney factor graph of Figure 3. The Bayesian network here does not denote a generative model; rather, it describes the generative process – the environmental process, including actions of the organism, that generated the sensory. The two graphs can be linked to depict the action-perception cycle: the policy half-edge of Figure 2 is coupled back to the generative process – namely, through the selection of an action that then determines state transitions. The causal processes in the world (inside the red box) generate a sequence of outcomes, which induce message passing and belief propagation, thus informing approximate posterior beliefs about policies. These policies determine the action to be selected, which in turn generates new outcomes, thereby closing the action-perception cycle in a circular causal embrace. The action that is selected by the process is the most probable one, given posterior beliefs about action sequences (aka policies). In this combined figure, we emphasise the circular causality of active inference by replacing the message labels with arrows. Source: From Friston, Parr, and de Vries (2017). Figure re-used from REF under the CC license.

The idea that generative models are structural representations rests on an oversimplified reading of these constructs, based in older Bayesian theories such as the Helmholtz machine (Dayan et al., 1995) and non-enactive appeals to variational Bayesian methods (Bishop, 2006), rather than on active inference under the FEP. In active inference, it is the recognition density that – through active inference – synchronises dynamically with the niche, and entails the generative model (examine Figure 4 again). The recognition density is encoded by the variables that are updated in active inference. The generative model does not coincide with these quantities, since it relates the quantities – the ones with the others – in an inferential net. There is no warrant, mathematically, for the claim that the generative model encodes semantic content or structural information. The generative model does *not* encode anything. It is *realised by* the statistical relations between states of interest. Instead, the expectations of the organism, as they figure under the generative model, are brought about by the organism in a kind of self-fulfilling prophecy through active inference.

Against representationalist interpretations, we emphasise the subtle, often missed point that the *generative model* is entailed by the *dynamics* (i.e., the *adaptive behaviour*) of the organism. The generative model manifests as a control system *that uses exploitable structural similarities encoded in the internal states of the organism*. It is not itself a representation, or anything like the *vehicle* of representational content. Conversely, the *recognition density* can be cast as having properties similar to those of a structural representation – in the sense that has been explored in recent literature on active inference and cognitive representations (e.g., Gładziejewski & Miłkowski, 2017; Kiefer & Hohwy, 2018). However, this only holds given that exploitable structural similarities are generated and maintained by active inference.

The structures that *do* encode exploitable structural similarities are the *internal states* of the Markov blanket, which parameterise a recognition density that the organism embodies, not the generative model. So, representationalists about generative models in active inference conflate quantities that should be held distinct – at least in the active inference framework. And this is the category error of these interpretations. Structural representationalism is correct in its ascription to organisms a set of internal (e.g., neural) structures that are apt to encode an exploitable structural resemblance, and which is used in the control of action.

The twist here is that this vindication of a representationalist sounding idea is accomplished by mobilising the resources of its traditional adversary, *enactivism*. Under the FEP, the organism’s internal states do indeed garner and encode exploitable, action-guiding dynamics about environmental states, as the representationalist maintains. However, they are established and maintained through active inference, that is, through patterns of adaptive action. And crucially, the generative model is nothing like these structures. It cannot be interpreted as representational, even in the weak sense of the proponents of structural representations.

The philosophical implication of conflating the generative process and the recognition density, and missing their role under the FEP, is to misunderstand the role of these constructs in the free energy formulation. A proper understanding of generative models under active inference, we have argued, is that they are ‘what an organism (normatively) expects’ and that they guide ‘what an organism is and does’. The generative model is therefore instantiated by expectations about how the world should be, where the expectations are conditioned on the adaptive preferences of the organism. This means that the generative model is realised by the embodied activity of an organism. It also suggests that the generative model is a control system *that uses exploitable structural similarities encoded in the internal states of the organism*. If this is correct, it is an outcome that allows us to accommodate key insights of representationalist views of active inference, without having to accept the claim that generative models are structural representations.

## 6. Concluding remarks

Although we have focused more narrowly on the active inference formulation in this article, our target and conclusions ultimately speak to much wider issues: the status of one of the most central (philosophical) concepts in the cognitive science –*representation*. Crucially, we have argued that, contrary to non-enactive, brain-centred Bayesian schemes such as predictive coding, the Bayesian brain and predictive processing, all of which have been articulated as vindicating the notion of structural representation, this particular reading turns out to be unjustified once we consider the mechanics of active inference under the free energy principle. Specifically, we have argued that the attempted vindication of structural representationalism in Bayesian cognitive science rests on a mistaken interpretation of the generative model and recognition density. Representationalists argue that generative models encode exploitable structural information about the world. Our analysis suggests that this is false. Indeed, in this article we sought to underpin the claim that generative models do not encode anything directly; they are rather expressed in embodied activity, and leverage information encoded in the recognition density (which is an approximate posterior belief or ‘best guess’). Assuming our conclusion is correct, our *enactive inference* proposal serves to free us from a standard, but flawed, philosophical assumption about the nature and explanatory basis of cognition and adaptive behaviour.

## Notes

1. In this article, we will use *recognition* density to mean an *approximate posterior* density or *variational* density that corresponds to a Bayesian or *posterior belief*– in the sense of (non-propositional) *belief* updating or *belief* propagation. Furthermore, we will read *model* as implicitly probabilistic – so that the recognition *model* becomes a recognition *density*.

2. Of note is that these beliefs do not (necessarily) pertain to propositional or subjective beliefs (Kirchhoff & Robertson, 2018). They are statistical constructs that bias action and perception, rather than beliefs in the philosophical sense, that is, of propositions with satisfaction or truth conditions. Technically, beliefs are used in the sense of Bayesian *belief* updating or *belief* propagation and refer to probability distributions whose parameters or sufficient statistics are associated with physical states.

3. Expected free energy is the same as variational free energy except that the expectation is taken under the posterior predictive density over future states and outcomes. Crucially, expected free energy is conditioned on a particular sequence of actions or a policy. This means, for every policy there is an expected free energy, enabling the selection of policies that minimise expected free energy or, more colloquially, uncertainty. Expected free energy is denoted by *G* in the legends for Figures 2 and 3.

4. Technically, the dynamics are a stochastic gradient flow on variational free energy that is a functional (i.e., a function of a function) of the generative model. This means, a sufficient description of any self-organising system is available in terms of *free energy gradients* that may, or may not, be expressed in terms of an explicit generative model.

5. See Kirchhoff and Kiverstein (2019, chapters 5 and 6) and Veissière, Constant, Ramstead, Friston, and Kirmayer (2020) for an account of how cultural practices can play a distinct role in organising the manner in which exchanges with the environment are structured, and consequently result in embodied action, including perceptual experiences.
